# Simplified Nonlinear Current-Mode Control of DC-DC Cuk Converter for Low-Cost Industrial Applications

**DOI:** 10.3390/s23031462

**Published:** 2023-01-28

**Authors:** Humam Al-Baidhani, Marian K. Kazimierczuk

**Affiliations:** 1 Department of Electrical Engineering, Wright State University, Dayton, OH 45435, USA; 2 Department of Computer Techniques Engineering, Faculty of Information Technology, Imam Ja’afar Al-Sadiq University, Baghdad 10011, Iraq

**Keywords:** analog control circuit, constant switching frequency, DC-DC Cuk converter, simplified nonlinear control, sliding-mode current control

## Abstract

This paper presents a robust nonlinear current-mode control approach for a pulse-width modulated DC-DC Cuk converter in a simple analog form. The control scheme is developed based on the reduced-state sliding-mode current control technique, in which a simplified equivalent control equation is derived using an averaged power converter model in continuous conduction mode. The proposed controller does not require an output capacitor current sensor and double proportional-integral compensators as in conventional sliding-mode current controllers; thus, the cost and complexity of the practical implementation is minimized without degrading the control performance. The simplified nonlinear controller rejects large disturbances, provides fast transient response, and maintains a constant switching frequency. The nonlinear control scheme is developed using an analog circuit with minimal added components, which is suitable for low-cost industrial applications. The control law derivation, control circuit design, controller gains selection, and stability analysis are provided. The proposed control methodology is verified via simulating the closed-loop nonlinear power converter model in MATLAB/SIMULINK under abrupt changes in load current and input voltage. The simulation results show that the proposed control scheme provides robust tracking performance, a low percentage overshoot, fast transient response, and a wide operating range. The maximum percentage overshoot and settling time of the closed-loop power converter response during line disturbance are 5.6% and 20 ms, respectively, whereas the percentage overshoot and settling time during load disturbance are 2.8% and 15 ms, respectively.

## 1. Introduction

Switched-mode DC-DC converters are key parts in modern power systems through which the DC input voltage is stepped up or down to meet the load voltage requirement. The DC-DC Cuk converter has been studied in the literature due to its excellent features, such as ripple-free input and output currents and wide conversion ratio [1]. However, the fourth-order dynamics of the DC-DC Cuk converter affect its bandwidth and transient response in the presence of line and load variations, which occur during normal operation condition. Hence, a robust control circuit is required to enhance the power converter response and track the desired reference voltage under large disturbances. In addition, the structure of the controller should be cost-effective and attractive for industrial applications [2]. In the literature, nonlinear control techniques have been proposed for such a type of power converter [3,4,5,6,7,8]. For instance, a cascaded system that incorporates a DC-DC boost converter supplying buck, Cuk, and SEPIC converters has been studied [3]. The nonlinear behavior of the cascaded system has been investigated with a two-loop controller, which contains inner current and outer voltage loops. In [4], a three-phase inverter-based Cuk converter with a proportional-integral (PI) controller has been introduced. However, the performance of such control techniques and their robustness under large line and load disturbances has not been discussed.

In [5,6,7,8,9,10,11], robust control methods have been studied with the DC-DC Cuk converter. For instance, a control algorithm based on fuzzy logic has been implemented with a photovoltaic (PV) system, in which a DC motor is fed via a Cuk power converter [5]. Another controller has been proposed in [6], using a passivity-based control method. A state-observer-based nonlinear sensorless controller has been applied to the boost and Cuk converters with unknown load conductance [7]. In [8], a down-sampled repetitive controller with a fractional-order phase-lead compensation has been utilized with the Cuk converter. Modern optimization algorithms have been integrated with the SMC method to control a hybrid interleaved boost-Cuk converter [9]. An ant colony-optimized maximum power-point tracking (MPPT) control system that employs a single Cuk converter as an impedance power adapter has been given in [10]. A fuzzy logic-based MPPT control technique for buck-boost, Cuk, SEPIC, and ZETA DC-DC converters has been studied for PV applications [11]. Despite the robust tracking performance of the aforementioned controllers, their practical implementation is complicated and requires expensive platforms such as dSPACE and DAQCard-6062E, which are not attractive for low-cost industrial applications.

A hybrid high-voltage gain converter has been proposed in [12] for PV applications, which incorporates a microcontroller to control the Cuk and boost converters. A commutation torque ripple suppression method based on a single-input dual-output Cuk converter has also been introduced [13], where PI current and speed controllers are implemented to regulate a brushless DC motor speed. Other research endeavors have applied the sliding-mode control (SMC) theory due to its robustness against large disturbances [14,15,16]. In [17], a neural-network-based SMC of a pulse-width modulated (PWM) DC-DC Cuk converter has been developed. A proportional, integral sliding-mode control (PI-SMC) method of the Cuk converter has been discussed in [18,19]. Previous research efforts have presented excellent control systems, but they fell short of providing a systematic design procedure for control circuits. A hysteresis modulation-based SMC method has been presented in [20] for the Cuk converter. However, the variable and high switching frequency inherited from the hysteresis modulation worsens EMI issues and complicates the required filter design [21]. Alternatively, ref. [22] has proposed a PWM-based reduced-state SMC scheme for a DC-DC Cuk converter, which maintains a constant switching frequency during sliding-mode operation. Although a systematic design procedure has been given in [22], the control scheme requires further simplification to minimize the control circuit complexity and achieve a practical implementation cost. Table 1 summarizes the modern control techniques that have been applied to the DC-DC Cuk converter in the previous literature.

In [23], a simplified double-integral sliding-mode current controller has been proposed for DC-DC boost converter in continuous conduction mode (CCM) using an analog control circuit. Motivated by [23], this paper aims to apply the simplified control design method to the DC-DC Cuk converter in CCM. The new control scheme does not require an output capacitor current sensor and double PI compensators, as in [22]. Thus, the simplified control law reduces the components count of the control circuit elements while maintaining a robust tracking performance. The contributions of this research are summarized as follows: (1) a simplified equivalent control law is derived using the averaged control-oriented model of a DC-DC Cuk converter. The control equation is developed based on the reduced-state PWM-based sliding-mode current control method; (2) the existence and stability conditions are derived to set the criteria for controller gains’ selection; (3) a systematic design procedure is introduced to construct the proposed control equation using a low-cost analog circuit; (4) the control design approach is verified via simulating the simplified nonlinear current-mode-controlled DC-DC Cuk converter model in MATLAB/SIMULINK.

The research work is organized as follows. The modeling of the DC-DC Cuk converter in CCM is given in Section 2. The design of simplified sliding-mode current control scheme is presented in Section 3. In Section 4, the design procedure of the proposed control scheme in analog form is introduced. Section 5 presents the flowchart of the proposed control system design. The simulation results are provided in Section 6, and the conclusions are summarized in Section 7.

## 2. Modeling of DC-DC Cuk Converter

### 2.1. Ideal Switched Model

**Figure 1:**
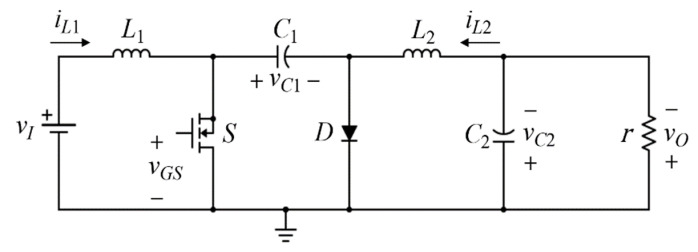
DC-DC Cuk converter circuit.

The circuit of the DC-DC Cuk converter is shown in Figure 1. It consists of a MOSFET switch *S*, a diode *D*, an input inductor $L_{1}$, an output inductor $L_{1}$, an energy transfer capacitor $C_{1}$, an output capacitor $C_{2}$, and a load resistor r. The instantaneous input and output voltage are defined as $v_{I}$ and $v_{O}$, respectively. The instantaneous input and output inductor currents are $i_{L1}$, and $i_{L2}$, respectively, whereas the instantaneous voltages across $C_{1}$ and $C_{2}$ are $v_{C1}$, and $v_{C2}$, respectively.

The switched-mode power converter is a variable structure system which depends on the control input value. In CCM, the ideal switched model of the DC-DC Cuk converter can be described as [1]

(1) $$\{\begin{array}{c} \frac{di_{L1}}{dt}=-\frac{1}{L_{1}}v_{C1}\bar{u}+\frac{1}{L_{1}}v_{I}\\ \begin{array}{c} \frac{di_{L2}}{dt}=\frac{1}{L_{2}}v_{C1}u-\frac{1}{L_{2}}v_{C2}\;\\ \frac{dv_{C1}}{dt}=\frac{1}{C_{1}}i_{L1}\bar{u}-\frac{1}{C_{1}}i_{L2}u\;\\ \frac{dv_{C2}}{dt}=\frac{1}{C_{2}}i_{L2}-\frac{1}{rC_{2}}v_{C2},\; \end{array} \end{array}$$

where the switching control input u takes the value 0 or 1, and $\bar{u}=1-u$.

### 2.2. Control-Oriented Model

The control-oriented model should be obtained to derive the simplified equivalent control law [2]. This model reflects the error dynamics of the DC-DC Cuk converter in CCM. Since the Cuk converter is a non-minimum phase system, a current-mode controller is required to enhance the closed-loop system response [1]. In sliding-mode current control, a reference current signal $i_{r}$ can be selected as

(2) $$i_{r}=K\left(V_{r}-\beta v_{O}\right),$$

where K, $V_{r}$, and β are a constant gain, a reference voltage, and a feedback network gain, respectively.

In order to derive the control-oriented model of the DC-DC Cuk converter, the control state variable can be selected as

(3) $$\{\begin{array}{c} x_{1}=i_{r}-i_{L1}\\ x_{2}=\int \left(V_{r}-\beta v_{O}\right)dt\\ \begin{array}{c} x_{3}=\int x_{1}dt\\ x_{4}=\int x_{2}dt. \end{array} \end{array}$$

The states $x_{1}$, $x_{2}$, $x_{3}$, and $x_{4}$ represent the input inductor current error, the integral of output voltage error, integral of $x_{1}$, and the integral of $x_{2}$, respectively. It should be noted that $x_{4}$ defines the double-integral term of the output voltage error, which helps to eliminate the steady-state error at the output voltage.

According to [1], the ideal switched power converter model in (1) can be reflected by the control state variables, which give

(4) $$\{\begin{array}{c} {\dot{x}}_{1}=-\frac{K\beta }{C_{2}}i_{C2}-\frac{v_{I}-v_{C1}\bar{u}}{L_{1}}\\ {\dot{x}}_{2}=V_{r}-\beta v_{O}\\ {\dot{x}}_{3}=i_{r}-i_{L1}\\ {\dot{x}}_{4}=\int \left(V_{r}-\beta v_{O}\right)dt. \end{array}$$

Based on the averaging theory, the averaged control-oriented model of the DC-DC Cuk converter is derived from [2], yielding

(5) $$\{\begin{array}{c} {\dot{\bar{x}}}_{1}=-\frac{K\beta }{C_{2}}{\bar{l}}_{C2}-\frac{{\bar{v}}_{I}-{\bar{v}}_{C1}{\bar{u}}_{e}}{L_{1}}\\ {\dot{\bar{x}}}_{2}=V_{r}-\beta {\bar{v}}_{O}\\ {\dot{\bar{x}}}_{3}=i_{r}-{\bar{l}}_{L1}\\ {\dot{\bar{x}}}_{4}=\int \left(V_{r}-\beta {\bar{v}}_{O}\right)dt. \end{array}$$

The averaged quantities of the output capacitor current, input inductor current, input voltage, and output voltage are represented as ${\bar{l}}_{C2}$, ${\bar{l}}_{L1}$, ${\bar{v}}_{I}$, and ${\bar{v}}_{O}$, respectively. The averaged quantity of the control input complement is defined as ${\bar{u}}_{e}=1-u_{e}$.

## 3. Simplified Nonlinear Current-Mode Control Design

### 3.1. Derivation of Equivalent Control Law

A switching control input is required to satisfy the hitting condition [1], which can be written as

(6) $$u=\frac{1}{2}\left[1+\mathrm{sign}\left(\psi \right)\right].$$

The sliding surface ψ is defined by

(7) $$\psi =\alpha_{1}x_{1}+\alpha_{2}x_{2}+\alpha_{3}x_{3}+\alpha_{4}x_{4},$$

where $\alpha_{1}$, $\alpha_{2}$, $\alpha_{3}$, and $\alpha_{4}$ represent the sliding coefficients.

Now, if the time derivative of the sliding surface is equated to zero, one obtains:

(8) $$\dot{\psi }=\alpha_{1}{\dot{x}}_{1}+\alpha_{2}{\dot{x}}_{2}+\alpha_{3}{\dot{x}}_{3}+\alpha_{4}{\dot{x}}_{4}=0.$$

According to the invariance conditions [21], the error dynamics of the DC-DC Cuk converter become zero at the sliding surface. The next step is to substitute the averaged control-oriented model into (8), which gives

(9) $$\alpha_{1}\left(-\frac{K\beta }{C_{2}}{\bar{l}}_{C2}-\frac{{\bar{v}}_{I}-{\bar{v}}_{C1}{\bar{u}}_{e}}{L_{1}}\right)+\alpha_{2}\left(V_{r}-\beta {\bar{v}}_{O}\right)+\alpha_{3}\left(i_{r}-{\bar{l}}_{L1}\right)+\alpha_{4}\left[\int \left(V_{r}-\beta {\bar{v}}_{O}\right)dt\right]=0.$$

The averaged capacitor current ${\bar{l}}_{C}$ is zero during steady-state operation [2]. Additionally, this component does not have a significant effect on the sliding-mode control process when compared to the other control state variables. Hence, if the term associated with ${\bar{l}}_{C}$ is set to zero, (9) becomes

(10) $$\alpha_{1}\left(-\frac{{\bar{v}}_{I}-{\bar{v}}_{C1}{\bar{u}}_{e}}{L_{1}}\right)+\alpha_{2}\left(V_{r}-\beta {\bar{v}}_{O}\right)+\alpha_{3}\left(i_{r}-{\bar{l}}_{L1}\right)+\alpha_{4}\left(\int \left(V_{r}-\beta {\bar{v}}_{O}\right)dt\right)=0.$$

Thus, the equivalent control law $u_{e}$ can be obtained by rearranging (10), which yields

(11) $$u_{e}=\frac{{\bar{v}}_{C1}-{\bar{v}}_{I}}{{\bar{v}}_{C1}}-\frac{L_{1}\alpha_{3}}{\alpha_{1}}\left(\frac{{\bar{l}}_{L1}}{{\bar{v}}_{C1}}\right)+\frac{L_{1}\left(K\alpha_{3}+\alpha_{2}\right)}{\alpha_{1}}\left(\frac{V_{r}-\beta {\bar{v}}_{O}}{{\bar{v}}_{C1}}\right)+\frac{L_{1}\alpha_{4}}{\alpha_{1}}\left[\frac{\int \left(V_{r}-\beta {\bar{v}}_{O}\right)dt}{{\bar{v}}_{C1}}\right].$$

The control law in (11) can also be written as

(12) $$u_{e}=\frac{{\bar{v}}_{C1}-{\bar{v}}_{I}}{{\bar{v}}_{C1}}-K_{L}\left(\frac{{\bar{l}}_{L1}}{{\bar{v}}_{C1}}\right)+K_{p}\left(\frac{V_{r}-\beta {\bar{v}}_{O}}{{\bar{v}}_{C1}}\right)+K_{i}\left[\frac{\int \left(V_{r}-\beta {\bar{v}}_{O}\right)dt}{{\bar{v}}_{C1}}\right].$$

The parameters of the equivalent control law $K_{L}$, $K_{p}$, and $K_{i}$ are defined by

(13) $${\left[\begin{array}{cc} \begin{array}{cc} K_{L} & K_{p} \end{array} & K_{i} \end{array}\right]}^{T}={\left[\begin{array}{cc} \begin{array}{cc} \frac{L_{1}\alpha_{3}}{\alpha_{1}} & \frac{L_{1}\left(K\alpha_{3}+\alpha_{2}\right)}{\alpha_{1}} \end{array} & \frac{L_{1}\alpha_{4}}{\alpha_{1}} \end{array}\right]}^{T}$$

which represent the inductor current, proportional, and integral gains, respectively.

**Figure 2:**
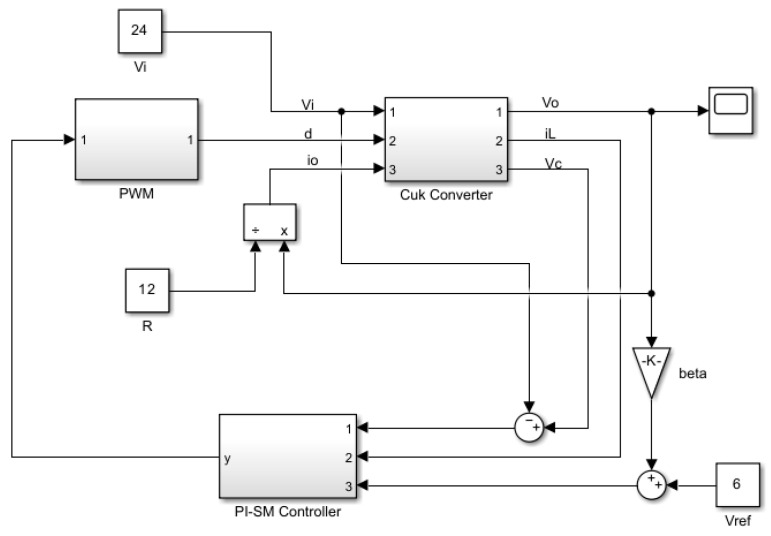
Block diagram of simplified double-integral sliding-mode current control of the DC-DC Cuk converter.

As reported in [1], the equivalent control law in (13) should be mapped onto a duty cycle d in order to be utilized via a pulse-width modulator. Hence, using the relationship $0<d=\frac{{\hat{u}}_{e}}{V_{T}}<1$, the simplified sliding-mode current control equation becomes

(14) $$\{\;\begin{array}{c} {\hat{u}}_{e}=\gamma \left({\bar{v}}_{C1}-{\bar{v}}_{I}\right)-\gamma K_{L}{\bar{l}}_{L1}+\gamma K_{p}\left(V_{r}-\beta {\bar{v}}_{O}\right)+\gamma K_{i}\int \left(V_{r}-\beta {\bar{v}}_{O}\right)dt\\ V_{T}=\gamma {\bar{v}}_{C1}, \end{array}$$

where $V_{T}$ is the peak ramp voltage of the pulse-width modulator. It should be noticed that the parameter γ in (14) is a scaling constant that is bounded between 0 and 1. This factor can be used to scale the controller gains and the peak ramp voltage down to fit the practical range of the analog components. The block diagram of the simplified current-mode controlled PWM DC-DC Cuk converter in CCM is depicted in Figure 2.

### 3.2. Existence and Stability Conditions

According to [1], if the local reachability condition $\underset{\psi \to 0}{\lim}\psi \dot{\psi }<0$ is satisfied, all the state trajectories of the closed-loop power converter remain within the vicinity of the sliding surface. For the given dynamics of the DC-DC Cuk converter, the existence condition can be written as

(15) $$\{\begin{array}{c} K_{p}\left(V_{r}-\beta v_{O}\right)-K_{L}i_{L1}+K_{i}x_{2}<v_{I}\\ K_{p}\left(V_{r}-\beta v_{O}\right)-K_{L}i_{L1}+K_{i}x_{2}>\left(v_{I}-v_{C1}\right). \end{array}$$

It can be seen that (15) depends on the controller gains $K_{L}$, $K_{p}$, and $K_{i}$. Hence, this condition set the first criterion for choosing proper control parameters for the simplified sliding-mode current controller.

The other criterion for choosing proper control parameters is the stability condition, which ensures that all the state trajectories converge towards the desired equilibrium point [22]. If the Cuk converter dynamics are set to zero, one can solve for the following equilibrium point:

(16) $$\{\begin{array}{c} V_{C1}=V_{I}+V_{o}\\ I_{L2}=\frac{V_{o}}{R}\\ I_{L1}=\frac{V_{o}^{2}}{V_{I}R} \end{array}$$

The steady-state quantities of the input voltage, output voltage, energy transfer capacitor voltage, input inductor current, output inductor current, and load resistor are defined as $V_{I}$, $V_{o}$, $V_{C1}$, $I_{L1}$, $I_{L2}$, and R, respectively. The stability of the closed-loop power converter dynamics around the desired equilibrium point must be analyzed.

The ideal model of the simplified sliding-mode current controlled PWM DC-DC Cuk converter in CCM can be obtained by substituting the equivalent control law into the ideal switched model given in (1), which yields

(17) $$\{\begin{array}{c} \frac{di_{L1}}{dt}=-\frac{1}{L_{1}}v_{C1}{\bar{u}}_{e}+\frac{1}{L_{1}}v_{I}\\ \begin{array}{c} \frac{di_{L2}}{dt}=\frac{1}{L_{2}}v_{C1}u_{e}-\frac{1}{L_{2}}v_{O}\;\\ \frac{dv_{C1}}{dt}=\frac{1}{C_{1}}i_{L1}{\bar{u}}_{e}-\frac{1}{C_{1}}i_{L2}u_{e}\\ \frac{dv_{C2}}{dt}=\frac{1}{C_{2}}i_{L2}-\frac{1}{rC_{2}}v_{O}. \end{array} \end{array}$$

where $u_{e}$ and ${\bar{u}}_{e}$ are the equivalent control law given in (12) and its complement, respectively.

The ideal closed-loop Cuk converter dynamics in (17) can be linearized around the equilibrium point, which gives

(18) $$[\begin{array}{c} {\dot{\tilde{x}}}_{1}\\ {\dot{\tilde{x}}}_{2}\\ {\dot{\tilde{x}}}_{3}\\ {\dot{\tilde{x}}}_{4}\\ {\dot{\tilde{x}}}_{5} \end{array}]=\begin{array}{cc} \left[\begin{array}{ccccc} j_{11} & 0 & 0 & j_{14} & j_{15}\\ j_{21} & 0 & j_{23} & j_{24} & j_{25}\\ j_{31} & j_{32} & 0 & j_{34} & j_{35}\\ 0 & 0 & j_{43} & j_{44} & 0\\ 0 & 0 & 0 & 1 & 0 \end{array}\right] & [\begin{array}{c} {\tilde{x}}_{1}\\ {\tilde{x}}_{2}\\ {\tilde{x}}_{3}\\ {\tilde{x}}_{4}\\ {\tilde{x}}_{5} \end{array}]. \end{array}$$

The states of the linearized model ${\tilde{x}}_{1}$, ${\tilde{x}}_{2}$, ${\tilde{x}}_{3}$, ${\tilde{x}}_{4}$, and ${\tilde{x}}_{5}$ represent ${\tilde{l}}_{L1}$, ${\tilde{v}}_{C1}$, ${\tilde{l}}_{L2}$, ${\tilde{v}}_{O}$, and $\int {\tilde{v}}_{O}dt$, respectively. On the other hand, the time derivative of the states ${\dot{\tilde{x}}}_{1}$, ${\dot{\tilde{x}}}_{2}$, ${\dot{\tilde{x}}}_{3}$, ${\dot{\tilde{x}}}_{4}$, and ${\dot{\tilde{x}}}_{5}$ represent ${\dot{\tilde{l}}}_{L1}$, ${\dot{\tilde{v}}}_{C1}$, ${\dot{\tilde{l}}}_{L2}$, ${\dot{\tilde{v}}}_{O}$, and $\frac{d}{dt}\int {\tilde{v}}_{O}dt$, respectively. The linearized model in (18) has been derived, assuming that $V_{i}=v_{I}$, R=r, $V_{r}=\beta V_{o}$, $I_{r}=I_{L1}=K\left(V_{r}-\beta v_{O}\right),$ $V_{C1}={\tilde{v}}_{C1}$, and $V_{O}={\tilde{v}}_{O}$. The terms $j_{11}$, $j_{14}$, $j_{15}$, $j_{21}$, $j_{23}$, $j_{24}$, $j_{25}$, $j_{31}$, $j_{32}$, $j_{34}$, $j_{35}$, $j_{43}$, and $j_{44}$ in the Jacobian matrix *J* are defined by

(19) $$J=\left[\begin{array}{ccccc} -\frac{K_{L}}{L_{1}} & 0 & 0 & -\frac{K_{p}\beta }{L_{1}} & -\frac{K_{i}\beta }{L_{1}}\\ \frac{K_{L}V_{o}}{V_{i}RC_{1}}+\frac{V_{i}}{C_{1}V_{C1}} & 0 & -\frac{V_{o}}{C_{1}\left(V_{i}+V_{o}\right)} & \frac{K_{p}\beta V_{o}}{V_{i}RC_{1}} & \frac{K_{i}\beta V_{o}}{V_{i}RC_{1}}\\ -\frac{K_{L}}{L_{2}} & \frac{1}{L_{2}} & 0 & -\left[\frac{K_{p}\beta }{L_{2}}+\frac{1}{L_{2}}\right] & -\frac{K_{i}\beta }{L_{2}}\\ 0 & 0 & \frac{1}{C_{2}} & -\frac{1}{RC_{2}} & 0\\ 0 & 0 & 0 & 1 & 0 \end{array}\right]$$

The characteristic equation of the Jacobian matrix is given by

(20) $$\lambda^{5}+P_{1}\lambda^{4}+P_{2}\lambda^{3}+P_{3}\lambda^{2}+P_{4}\lambda +P_{5}=0,$$

where the parameters of the characteristic equation $P_{1}$, $P_{2}$, $P_{3}$, $P_{4}$, and $P_{5}$ are

(21) $$\left\{ \begin{array}{c} P_{1}=-(j_{11}+j_{44})\\ P_{2}=j_{11}j_{44}-j_{23}j_{32}-j_{34}j_{43}\\ P_{3}=-j_{35}j_{43}+j_{11}j_{23}j_{32}+j_{11}j_{34}j_{43}-j_{14}j_{31}j_{43}+j_{23}j_{32}j_{44}-j_{24}j_{32}j_{43}\\ P_{4}=j_{11}j_{35}j_{43}-j_{15}j_{31}j_{43}-j_{25}j_{32}j_{43}-j_{11}j_{23}j_{32}j_{44}+j_{11}j_{24}j_{32}j_{43}-j_{14}j_{21}j_{32}j_{43}\\ P_{5}=j_{11}j_{25}j_{32}j_{43}-j_{15}j_{21}j_{32}j_{43}. \end{array}\right.$$

The stability of the linearized closed-loop DC-DC Cuk converter dynamics around the equilibrium point is guaranteed if all Eigen values of the Jacobian Matrix have a negative real part. Based on the Routh–Hurwitz stability criterion, the stability conditions can be derived from (20), yielding

(22) $$\left\{ \begin{array}{c} P_{1}>0\\ P_{2}>\frac{P_{3}}{P_{1}}\\ P_{3}>\frac{P_{1}P_{4}-P_{5}}{P_{2}-\frac{P_{3}}{P_{1}}}\\ P_{4}>\frac{P_{4}[\frac{P_{5}}{P_{1}}+P_{2}(P_{2}-\frac{P_{3}}{P_{1}})]}{2P_{5}-P_{1}P_{4}+P_{3}(P_{2}-\frac{P_{3}}{P_{1}})}\\ P_{5}>0 \end{array}\right.$$

Thus, (22) can be solved numerically to determine the controller gains that guarantee the stability of the system.

The proportional, integral, and input inductor current gains of the simplified sliding-mode current controller should be selected such that both the existence and stability conditions are satisfied. For the DC-DC Cuk converter parameters given in Table 2 [22], the proposed equivalent control Equation is

(23) $$\{\;\begin{array}{c} {\hat{u}}_{e}=0.1\left({\bar{v}}_{C1}-{\bar{v}}_{I}\right)-0.4{\bar{l}}_{L1}+1.0\left(V_{r}-\beta {\bar{v}}_{O}\right)+170\int \left(V_{r}-\beta {\bar{v}}_{O}\right)dt\\ V_{T}=6\;V. \end{array}$$

**Figure 3:**
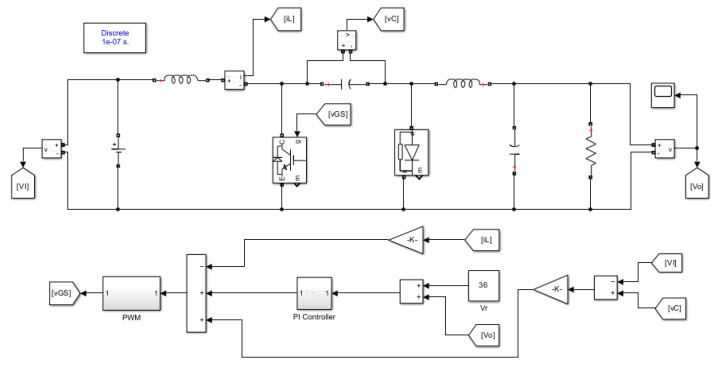
MATLAB/SIMULINK model of the simplified double-integral sliding-mode current control of the DC-DC Cuk converter.

The controller gains $K_{L}$, $K_{p}$, and $K_{i}$ are chosen according to the existence and stability conditions to be 0.4, 1, and 170, respectively. The peak ramp voltage $V_{T}$ is 6 V. In (23), it can be noticed that the scaling factor γ in this control equation is set to 0.1 to reduce the peak ramp voltage $V_{T}$ of the pulse width modulator and the controller gains to reasonable values that fit the analog control circuit implementation. Obviously, both ${\hat{u}}_{e}$ and $V_{T}$ are multiplied by this factor to maintain a consistent relationship between the equivalent control law and the ramp voltage. The MATLAB/SIMULINK model of the simplified sliding-mode current controlled PWM DC-DC Cuk converter is depicted in Figure 3.

## 4. Realization of Simplified Current-Mode Control Circuit

**Figure 4:**
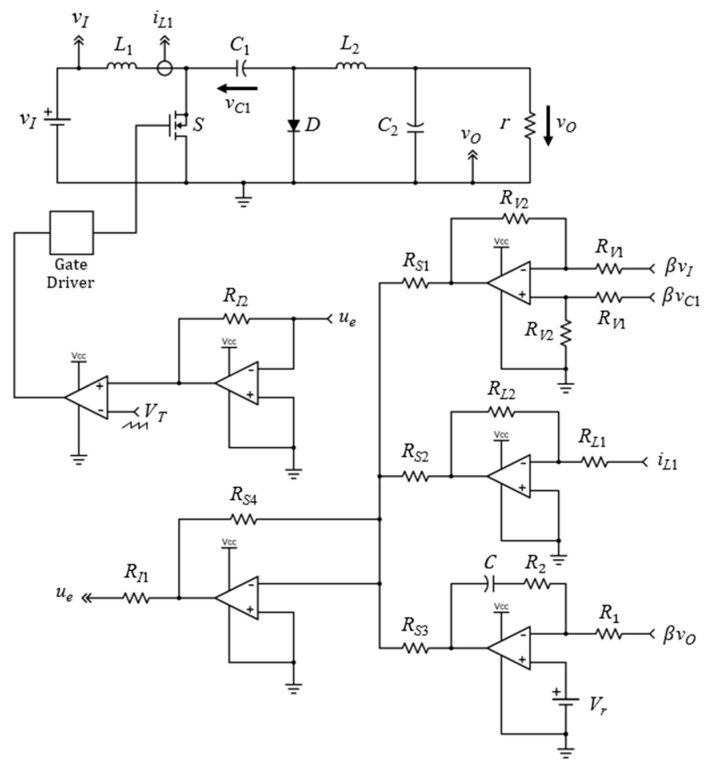
Schematic of proposed control circuit of the PWM DC-DC Cuk converter.

In this section, the design procedure of the simplified analog sliding-mode current control circuit of PWM DC-DC Cuk converter is introduced. The schematic of the closed-loop power converter circuit is shown in Figure 4. The proposed control equation in (23) can be constructed in a simple analog form, such that the implementation cost is minimized without compromising the tracking performance and robustness against large disturbances.

As shown in Figure 4, the proposed control circuit is built using op-amps, resistors, and a capacitor. The design procedure and the selection of components are given below.

- Voltage sensor gain β: The desired output voltage in this research is 36 V. Hence, if the reference voltage $V_{r}$ is set to 6 V, then the voltage sensor gain $\beta =\frac{V_{r}}{V_{o}}=\frac{1}{6}$.
- Summing and inverting op-amps: The resistors $R_{S1}$, $R_{S2}$, $R_{S3}$, and $R_{S4}$ for the summing op-amp, and $R_{I1}$ and $R_{I2}$ for the inverting op-amp, can be set to 5.1 kΩ.
- Pulse-width modulator: The peak ramp voltage $V_{T}$ and the switching frequency $f_{s}$ of the pulse-width modulator are chosen to be 6 V and 200 kHz, respectively.
- Proportional gain: As detailed in [24], the proportional gain $K_{p}$ of the PI compensator in the analog control circuit is defined as $K_{p}=\frac{R_{2}}{R_{1}}$. Thus, if the gain $K_{p}$ is set to 1, as in (23), then the resistors $R_{1}$ and $R_{2}$ are 5.1 kΩ.
- Integral gain: According to [24], the integral gain is $K_{i}=\frac{1}{R_{1}C}$. Since the gain $K_{i}$ and the resistor $R_{1}$ are chosen to be 170 and 5.1 kΩ, respectively, the capacitor *C* is 5.6 µF.
- Input inductor gain: The gain of the input inductor $K_{L}=\frac{R_{L2}}{R_{L1}}$. If the gain value is set to 0.4 and the resistor $R_{L1}$ is 10 kΩ, then the value of the resistor $R_{L2}$ is 4 kΩ.
- Differential amplifier: The resistors $R_{V1}$ and $R_{V2}$ of the differential amplifier in the control circuit are set to 10 kΩ.

It is worth mentioning that the design procedure given above can help the researchers and power electronics engineer to implement the simplified sliding-mode current control equation of the PWM DC-DC Cuk converter using a low-cost analog circuit. In addition, the choice of the controller gains $K_{p}$, $K_{i}$, and $K_{L}$ is not unique. The selected values can be adjusted to fit the practical range of the analog components. However, the choice of the simplified control equation parameters must satisfy the existence and stability conditions to ensure a proper sliding-mode control operation [2].

## 5. Framework of Developing Simplified Nonlinear Current-Mode Control Circuit

**Figure 5:**
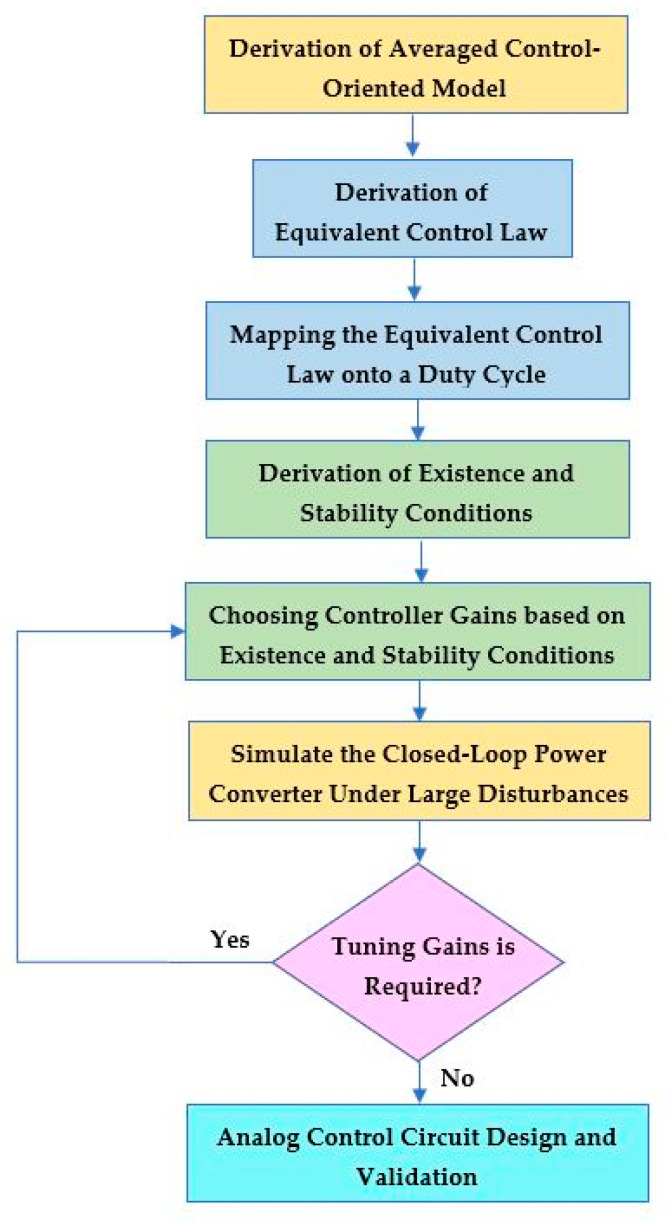
Development of proposed control circuit of PWM DC-DC Cuk converter.

A flowchart that shows the development of the double-integral sliding-mode current control circuit of the PWM DC-DC Cuk converter in CCM is depicted in Figure 5. It summarizes the design process that has been described in Section 2, Section 3 and Section 4.

The first step is the modeling of the power converter. The averaged control-oriented model is developed based on the voltage and current error dynamics of the power converter. The choice of the control state variables is made to drive the error signals of the output voltage and input inductor current to zero. The control state variables are then derived with respect to time, and averaged to obtain the averaged control-oriented model.

The second step is the derivation of the simplified equivalent control law based on the invariance conditions. The sliding surface dynamic is equated to zero to solve for the equivalent control law *u_e_*. Since the control equation is continuous, it should be mapped onto a duty cycle *d* so that it may be implemented via a pulse-width modulator. Next, the existence and stability conditions are derived based on the local reachability condition and Routh–Hurwitz stability criterion, respectively. The controller gains must be chosen such that both existence and stability conditions are satisfied to ensure a proper sliding-mode control operation.

The closed-loop power converter model is then simulated in MATLAB/SIMULINK, considering various operation conditions and large disturbances to validate the control design methodology. If the desired transient characteristics are not met, then another set of controller gains should be selected according to the existence and stability conditions. The final step is to convert the simplified current-mode control equation to an analog circuit for further testing and validation before building the experimental prototype.

## 6. Results and Discussion

### 6.1. Steady-State Performance

The simplified nonlinear current-mode controller of the PWM DC-DC Cuk converter in CCM is simulated using MATLAB/SIMULINK. The parameters of the switched-mode power converter are defined in Table 2, while the proposed control scheme is constructed as depicted in Figure 3. The closed-loop DC-DC converter is operated at 24 V input voltage and 20 Ω load resistor under steady-state conditions. Since the PWM DC-DC Cuk converter provides a negative output voltage, the desired output voltage is maintained at −36 V.

**Figure 6:**
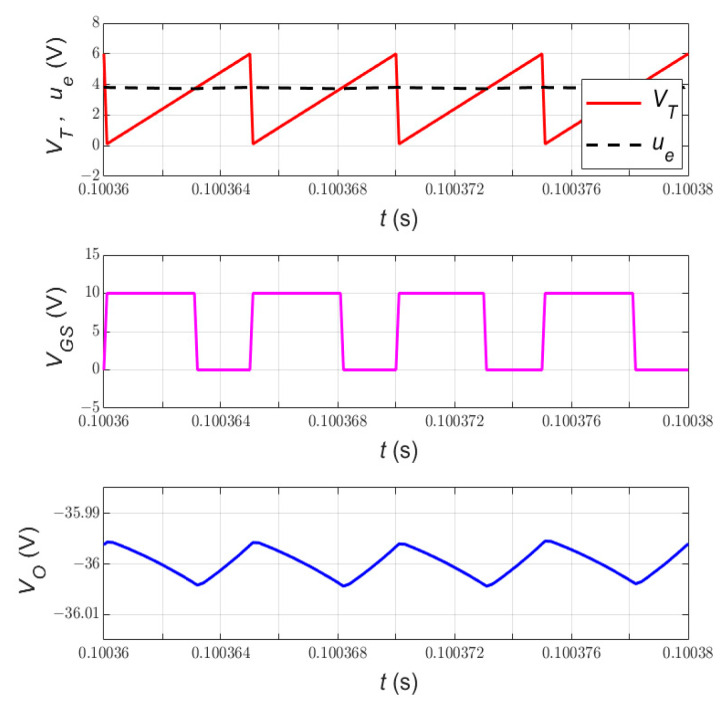
Steady-state waveforms of the simplified nonlinear current-mode controlled DC-DC Cuk converter at 24 V input voltage and 20 Ω load resistance. The figure shows the ramp voltage $V_{T}$, control voltage $u_{e}$, gate-to-source voltage $V_{GS}$, and output voltage $V_{O}$.

The steady-state waveforms of the ramp voltage $V_{T}$, control voltage $u_{e}$, gate-to-source voltage $V_{GS}$, and output voltage $V_{O}$ are presented in Figure 6. As shown in $V_{GS}$ waveform, the switching frequency is maintained at 200 kHz. The constant switching frequency in sliding-mode control performance is important to reduce both EMI noise issues and filter design complexity [21]. This is the main advantage of designing the sliding-mode controller via a pulse-width modulator.

It can also be noticed that the output voltage of the simplified current-mode controlled DC-DC Cuk converter tracks the desired trajectory precisely at −36 V. It is known that the tracking performance of the SMC scheme is degraded if it is used via a pulse-width modulator [1]. This is attributed to the operation of the SMC system at a constant and low switching frequency. Clearly, the pulse-width modulator works against the nature of the sliding-mode control theory, which requires variable and high switching to eliminate the steady-state error. However, the steady-state error has been eliminated thanks to the double-integral state variable in the control-oriented model.

### 6.2. Large Disturbance Rejection Capability

**Figure 7:**
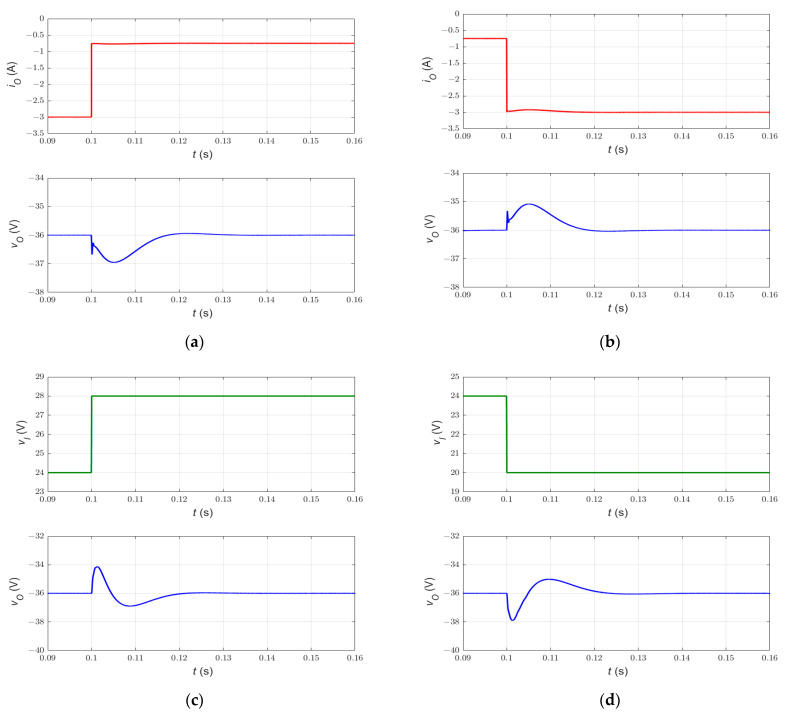
Transient response of simplified double-integral sliding-mode current control of the PWM DC-DC Cuk converter. The response during step (**a**) decrease and (**b**) increase in load current $i_{O}$. The response during step (**c**) increase and (**d**) decrease in input voltage $v_{I}$.

The simplified nonlinear current-mode control of PWM DC-DC Cuk converter has been tested under abrupt changes in input voltage $v_{I}$ and load current $i_{O}$. Figure 7 shows the tracking performance of the closed-loop power converter during line and load disturbances. The transient response characteristics of the proposed control system are summarized in Table 3.

Figure 7a,b show the system response when the load resistance r increases from 12 Ω to 48 Ω and decreases from 48 Ω to 12 Ω at *t* = 0.1 s, respectively. It can be seen that the output voltage waveform exhibits 2.8% percentage overshoot/undershoot and 15 ms settling time (1% criterion). On the other hand, Figure 7c,d show the response during the line disturbance, where the input voltage $v_{I}$ increases from 24 V to 28 V and decreases from 24 V to 20 V, respectively. The results show that the output voltage waveform has a percentage overshoot/undershoot of 5.6% and a settling time of 30 ms (1% criterion). Obviously, the simulation results of the simplified nonlinear controller show consistent dynamical response, large disturbance rejection capability, and zero steady-state error.

Furthermore, it can be observed that the proposed control method handles the non-minimum phase property of the DC-DC Cuk converter. In other words, the current-mode control method yields fast response and enhanced transient characteristics. On the contrary, the voltage-mode control of systems with a right-half plane zero results in a slow system response and a narrow bandwidth [25]. Hence, the simplified nonlinear current-mode controller enjoys simplicity in control circuit structure, robustness against large disturbances, and suitability for a non-minimum phase power converter.

### 6.3. Comparison with Classical Sliding-Mode Current Controller

The proposed sliding-mode current control of the PWM DC-DC Cuk converter is compared with the classical sliding-mode current control method in [1,22]. The tracking performance and disturbance rejection capability have been investigated. The classical control equation $u_{e}^{*}$ of the PWM DC-DC Cuk converter parameters given in Table 1 is designed according to [1,22], and expressed as

(24) $$\left\{ \begin{array}{c} u_{e}^{*}=0.5\left(V_{r}-\beta v_{O}\right)+100\int \left(V_{r}-\beta v_{O}\right)dt+0.5\left[1.0\left(V_{r}-\beta v_{O}\right)-0.4i_{L1}\right]\\ \;+\;0.5\int \left[50\left(V_{r}-\beta v_{O}\right)-0.4i_{L1}\right]-0.15i_{C2}+0.1\left(v_{C1}-v_{I}\right)\\ V_{T}=6\;V. \end{array}\right.$$

The control parameters in (24) have been selected to satisfy the existence and stability conditions and achieve the desired response specifications. It is also assumed that the peak ramp voltage $V_{T}$ is constant, as in (23).

Comparing the classical control equation $u_{e}^{*}$ in (24) to the simplified control equation ${\hat{u}}_{e}$ in (23), it can be noticed that the structure of proposed controller ${\hat{u}}_{e}$ is simpler than that of $u_{e}^{*}$. The classical control equation contains two PI compensators and a capacitor current sensor, which complicate the control circuit structure and cost of practical implementation. On the contrary, the simplified control equation does not require a capacitor current sensor and contains one PI compensator. Clearly, the simplification in the control scheme reduces the electronic components’ count and the implementation cost of the analog control circuit.

**Figure 8:**
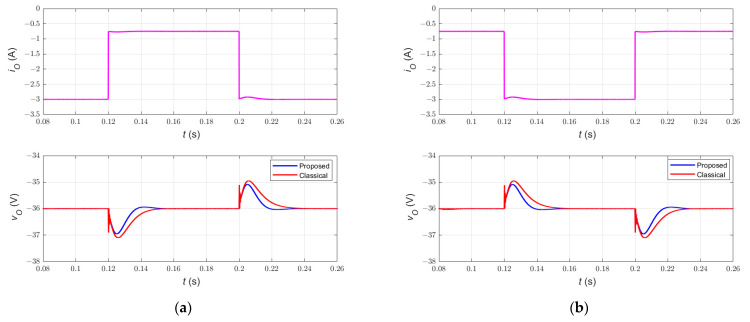

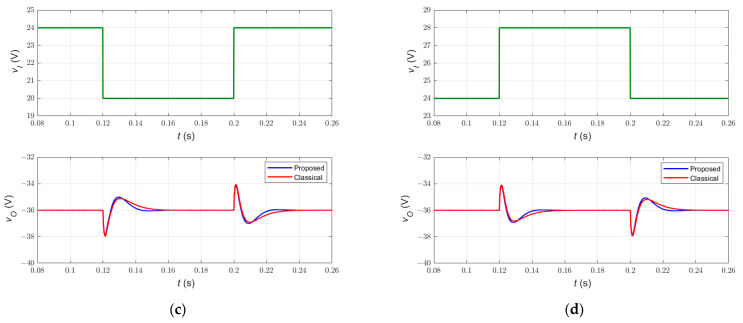
Comparison between the response of the proposed and classical double-integral sliding-mode current control of the PWM DC-DC Cuk converter under line and load disturbances. The response during step (**a**) decrease and (**b**) increase in load current $i_{O}$. The response during step (**c**) decrease and (**d**) increase in input voltage *v_I_*.

The performance of the two nonlinear control methods has also been investigated. Figure 8a,b show the closed-loop DC-DC Cuk converter response during abrupt changes in load current. In Figure 8a, a step change in load current $i_{O}$ from 3 A to 0.7 A occurs at *t* = 0.12 s, then $i_{O}$ returns back to 3 A at *t* = 0.2 s. In Figure 8b, however, the load current increases from 0.7 A to 3 A at *t =* 0.12 s, then decreases from 3 A to 0.7 A at *t* = 0.2 s. It can be noticed that both controllers track the desired trajectory and reject the load disturbance effect. Additionally, the response of the simplified and classical control methods exhibits a settling time of 15 ms and 22 ms (1% criterion), respectively. Moreover, the simplified and classical control methods show a percentage overshoot/undershoot of 2.8% and 3%, respectively.

In Figure 8c,d, the tracking performance of the two controllers under large line disturbance has been investigated. Figure 8c shows the response when the line voltage decreases from 24 V to 20 V at *t* = 0.12 s, then returns to 24 V at *t* = 0.2 s. On the other hand, Figure 8d shows the response when the line voltage increases from 24 V to 28 V at *t =* 0.12 s, then decreases from 28 V to 24 V at *t* = 0.2 s. It can be noticed that the simplified and classical double-integral sliding-mode current controllers exhibit a percentage overshoot/undershoot of 5.6%. However, the settling time of the simplified and classical controllers is 20 ms and 22 ms (1% criterion), respectively.

Table 4 shows a comparison between the simplified and classical double-integral SMC schemes of DC-DC Cuk converter. Notably, the two control methods track the desired trajectory under large disturbances and show about the same percentage overshoot/undershoot. However, the simplified DI-SMC scheme has a faster dynamical response compared to that of the classical DI-SMC scheme developed in [1,22]. More importantly, the superiority of the proposed nonlinear control method is shown in the simplicity of the control equation, which yields a cost-effective robust control circuit for the DC-DC Cuk converter.

## 7. Conclusions

A simplified nonlinear current-mode controlled PWM DC-DC Cuk converter has been presented using an analog control circuit. The proposed control scheme has been designed based on the sliding-mode control theory, through which a new equivalent control equation has been derived. A double-integral state variable has been included in the derivation of the control-oriented model of the power converter to eliminate the steady-state error at the output voltage. The non-minimum phase property of the DC-DC Cuk converter has been accommodated for by using the current-mode control method, where the input inductor current tracks a reference current to ensure a fast and consistent dynamical response. The existence and stability conditions have been given to set the criteria for the control parameters’ selection. In addition, a systematic design procedure has been presented to construct the proposed control law in a simple analog circuit using few op-amps and passive components. The proposed control scheme contains a single proportional-integral compensator and does not include the output capacitor current sensor and the relevant electronic components. Thus, the complexity and cost of practical implementation has been reduced significantly compared to previous nonlinear control methods. The tracking performance, transient response characteristics, and large disturbance rejection have been compared for the proposed and classical nonlinear current-mode control of a PWM DC-DC Cuk converter. It has been confirmed that both control schemes maintain robust tracking performance under large line and load disturbances. The simplified nonlinear control method yields a simple, robust, and cost-effective analog control circuit that is suitable for industrial applications. Future work will be to develop a PCB prototype for the proposed control circuit and validate the design approach experimentally. Further research and analysis can also be carried out to apply the simplified control method to other DC-DC power converters, such as Zeta and SEPIC converters.

## Figures and Tables

**Table 1:** Summary of modern control applications of a DC-DC Cuk converter.

Control Method	Advantages	Drawbacks	References
Fuzzy logic-based control	- Fast tracking performance - Programmability - Robustness	- Design complexity - Implementation cost	[5,11]
Passivity-based control			[6]
State observer-based control	- Fast response - Time-varying load estimation	- Design complexity - Implementation cost	[7]
Repetitive control	- Short computation time - Improved tracking performance	- Design complexity - Implementation cost	[8]
Modern optimization control	- Reduced input current ripple - Improved performance	- Implementation of control algorithm was not provided	[9]
Ant colony-based control	- Fast response - Improved performance	- Design complexity - Implementation cost	[10]
Neural network-based SMC	- Analog control circuit - Robustness and stability	- Design procedure of control circuit was not provided	[17]
Proportional-integral SMC	- Large-signal stability - Robustness - Accurate tracking	- Realization of control circuit was not provided	[18,19]
Hysteresis-modulated SMC	- Simplicity - Cost-effective	- Variable and high switching frequency (EMI issues)	[20]
PWM double-integral SMC	- Constant switching frequency - Analog control circuit	- It requires capacitor current sensor and two PI controllers	[22]

**Table 2:** The parameters of the DC-DC Cuk converter.

Description	Parameter	Value
Input inductance	$L_{1}$	400 µH
Internal resistance of $L_{1}$	$r_{L1}$	0.12 Ω
Output inductance	$L_{2}$	200 µH
Internal resistance of $L_{2}$	$r_{L2}$	0.12 Ω
Energy transfer capacitance	$C_{1}$	2200 µF
ESR of $C_{1}$	$r_{C1}$	25 mΩ
Output filter capacitance	$C_{2}$	230 µF
ESR of $C_{2}$	$r_{C2}$	25 mΩ
Input voltage	$v_{I}$	24 V
Output voltage	$v_{O}$	36 V
Load resistance	r	(12–48) Ω
Switching frequency	$f_{s}$	200 kHz

**Table 3:** The transient response characteristics of the proposed nonlinear controlled PWM DC-DC Cuk converter in CCM.

Line/Load Disturbance	Percentage Peak Overshoot/Undershoot (%)	Settling Time (ms)
$\Delta i_{O}\;\to \;\;3\;A\;\mathrm{to}\;0.75\;A$	2.8	15
$\Delta i_{O}\;\to \;\;0.75\;A\;\mathrm{to}\;3\;A$	2.8	15
$\Delta v_{I}\;\to \;24\;V\;\mathrm{to}\;20\;V$	5.6	20
$\Delta v_{I}\;\to \;24\;V\;\mathrm{to}\;28\;V$	5.6	20

**Table 4:** Comparison between classical and simplified DI-SMC of DC-DC Cuk converter.

Control Method	Required Sensors	PI Compensators	Characteristics during Load Disturbance	Characteristics during Line Disturbance
Classical DI-SMC	*v_O_*, *v_I_*, *i_L_*_1_, *v_C_*_1_, *i_C_*_2_	Dual	*PO* = 3%, *t_s_* = 22 ms	*PO* = 5.6%, *t_s_* = 22 ms
Simplified DI-SMC	*v_O_*, *v_I_*, *i_L_*_1_, *v_C_*_1_	Single	*PO* = 2.8%, *t_s_* = 15 ms	*PO* = 5.6%, *t_s_* = 20 ms

## Data Availability

Not applicable.
